# Engagement in child psychiatry department appointments: An analysis of electronic medical records in one safety-net hospital in New England, USA

**DOI:** 10.1177/13558196241311712

**Published:** 2025-01-16

**Authors:** Yesenia Aguilar Silvan, Lisa R Fortuna, Andrea E Spencer, Lauren C Ng

**Affiliations:** 1 Clinical Psychology Doctoral Student and Researcher, Department of Psychology, 8783 University of California Los Angeles, Los Angeles, CA, USA; 2 Professor and Chair, Department of Psychiatry and Neurosciences, School of Medicine, 378743 University of California, Riverside, CA, USA; 3 Associate Professor and Vice Chair for Research, Pritzker Department of Psychiatry and Behavioral Health, 2429 Ann and Robert H Lurie Children’s Hospital of Chicago, Chicago, IL, USA; 4 Assistant Professor, Department of Psychology, 8783 University of California Los Angeles, Los Angeles, CA, USA

**Keywords:** engagement, psychiatry, referrals, disparities

## Abstract

**Objective:**

This study examined whether being scheduled in a screening clinic versus scheduled directly with a long-term provider to conduct a mental health intake (MHI) is associated with engagement in child psychiatry services in New England, USA.

**Method:**

We used electronic medical record data from one safety-net hospital serving a predominantly low-income and minoritised population. The study sample included 815 youths aged 0 to 25 years, referred or scheduled for a MHI between 1 January 2016 and 31 December 2016. We used chi-square and t-tests to examine the association between referral pathways and engagement, logistic regression to understand the relationship between youth’s socio-demographic characteristics and referral pathways, and logistic and Poisson regressions to assess potential moderating effects of socio-demographic characteristics on engagement.

**Results:**

The mean age of the study population was 12 years; 46% were female, and the majority had public health insurance (84%) and lived in high social vulnerability areas (65%). Less than half of the youth attended the first scheduled MHI visit. Those scheduled with the screening clinic were less likely than those scheduled with the provider to ever attend a MHI appointment. Spanish-speakers were more likely to be directly scheduled with a provider (Odds Ratio, OR 0.48; 95% CI: 0.32, 0.73), while those with public health insurance were more likely to be scheduled with the screening clinic (OR 0.56; 95% CI: 0.43, 0.96). Spanish-speaking status and areas social vulnerability scores moderated the relationship between the referral pathway and engagement in psychiatric appointments.

**Conclusions:**

The study highlights the need for psychiatric services to evaluate how MHI referral procedures may mitigate barriers to care and facilitate engagement for youth at high risk of not attending psychiatric service appointments.

## Introduction

Poor attendance is a well-documented challenge in child psychiatric services.^1,2^ Approximately one third of youth referred for specialty care do not attend their first-session appointment^3^; average attendance is around four scheduled visits,^4^ or lower among minoritised youth.^5^

Mental health intake (MHI) procedures, which include initial clinical interviews, intake interviews, or clinical diagnostic interviews, are meant to help clients initiate care in child psychiatry services,^6–8^ although they may inadvertently perpetuate lower treatment engagement. While MHI procedures are common in psychiatry practice,^9–11^ there is a lack of empirical evidence supporting their use,^9,12,13^ with limited understanding of how these procedures influence engagement in child psychiatry services.^2^

**Figure 1.**
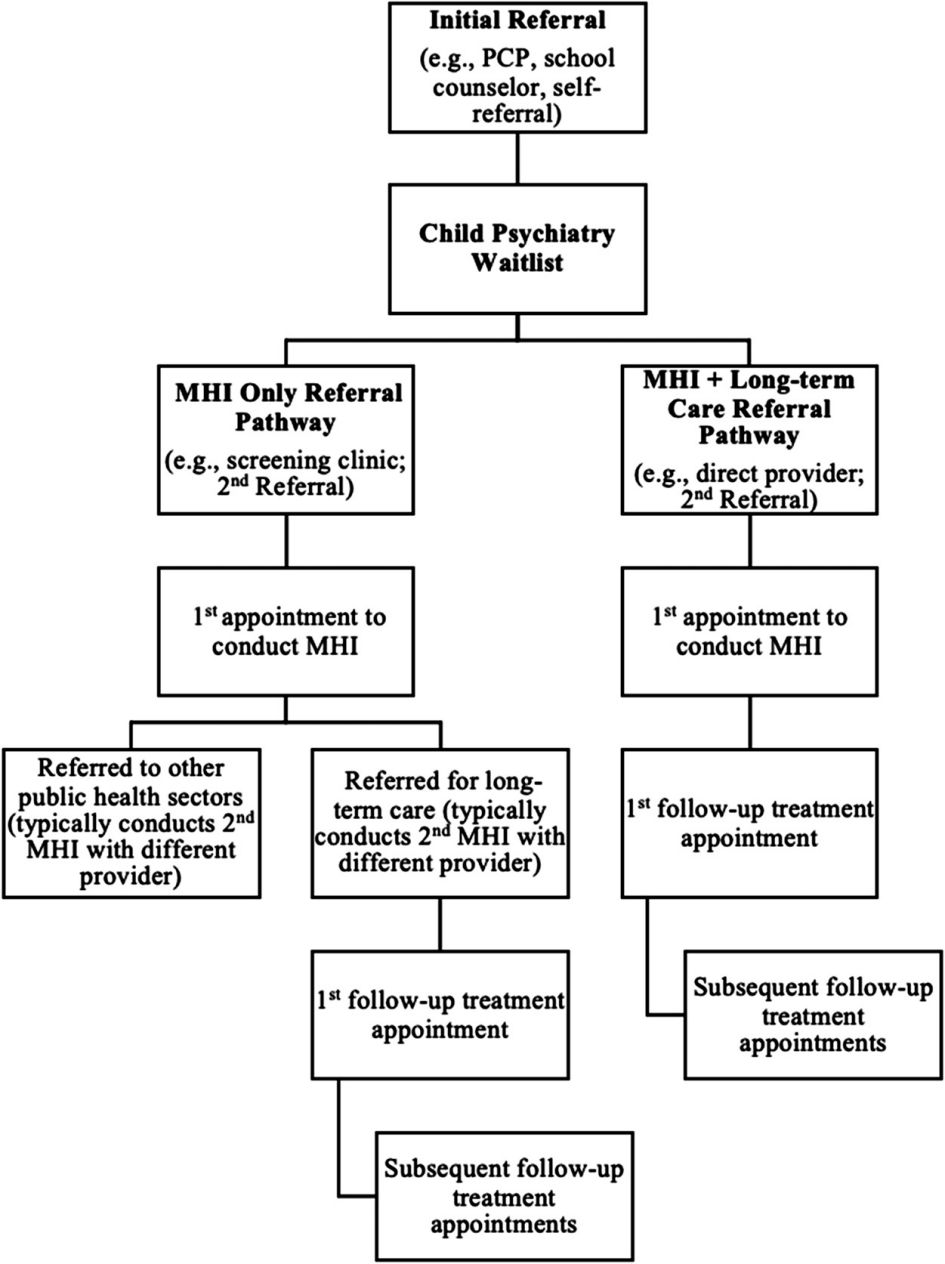
Types of referral pathways in child psychiatry services.

There are two common MHI pathways. In the MHI-Only Referral Pathway (MHI only) a client completes the MHI with a screening clinic or provider who will then refer them to a second provider for long-term care. In the MHI + Long-Term Care Referral Pathway (MHI + long-term care), the provider that conducts the MHI also delivers long-term care (Figure 1). There is strong evidence to suggest that clients who continue with their MHI providers for long-term care benefit from care continuity, building upon the established therapeutic relationship formed during the MHI.^12–15^ Conversely, the MHI only referral pathway may disrupt care, leading to perceptions of uncoordinated treatment.^16^

Youth may disclose sensitive information, including about their cultural background, immigration history, discrimination, racism, and trauma, for the first time during the MHI,^17^ requiring a ‘leap of faith’ and trust in the provider. Entering the service through the MHI only pathway means that clients will have to re-share their histories with different providers. This may lead to people discontinuing their care and increase mistrust, worsening treatment outcomes and attendance in mental health treatment.

There is some evidence that the MHI only pathway can exacerbate existing barriers to care for culturally and linguistically diverse youth and those facing high levels of social vulnerability. They often already encounter barriers such as resource inequality and discriminatory practices,^6,8,13,14,16,18^ making it even more difficult for them to engage in care. They may also be more likely to be triaged to the MHI only pathway due to well-documented systematic disparities in care pathways,^19^ with underserved clients often not referred to the best treatment options.^18^ Young people are more likely to engage in mental health treatment when they establish a direct connection with a provider for long-term care,^15^ suggesting that the MHI + long-term care referral pathway may improve attendance in child psychiatry services compared to the MHI only referral pathway.

The MHI only and MHI + long-term care referral pathways are established procedures in clinical practice but there is a lack of standardisation,^6,13^ and limited understanding of their relative impacts.^1^ In this study we examined the potential effects of these referral pathways on engagement in psychiatric appointments in a large safety-net hospital in the USA. We hypothesised that the MHI only referral pathway would be associated with worse rates of initial, delayed, and sustained engagement in psychiatry department services compared to the MHI + long-term care pathway. Based on research examining systemic racism in health care in the USA,^18^ we further hypothesised that youth who are non-English speakers, have public insurance, and face high levels of social vulnerability, would be more likely to be referred to the MHI only pathway compared to the MHI + long-term care pathway. Finally, we hypothesised that socio-demographic characteristics will moderate the relationship between referral pathways and engagement, that is, Spanish speakers, those with public insurance, and with high levels of social vulnerability who are referred to the MHI only pathway will have poorer initial and sustained engagement as well as more delayed engagement than their counterparts.

## Methods

The study was set in the largest safety-net hospital in New England in the USA. In 2016, over half (54%) of the patient population served by the hospital were from racial/ethnic minoritised communities, some 46% spoke a non-English language at home, and 75% had government insurance (e.g., Medicaid).^20^ Most patients reported that poverty was a major stressor in their lives.

### Study sample

We used deidentified electronic medical records to identify young people aged 0–25 years who were referred or scheduled for a MHI at the safety-net hospital’s Child & Adolescent Outpatient Psychiatry Center between 1 January 2016 to 31 December 2016. This period was selected following standard clinical referral procedures to account for seasonal variation in scheduled visits (e.g., due to the school calendar). Of all youths seen during that period (*N* = 1461), 815 met our inclusion criteria; 646 were excluded because they were existing patients continuing care from the previous year. Data extracted from the final sample included demographics, referrals, and visits (scheduled, rescheduled, cancelled, and attended). All psychiatry department visit types were included; thus we considered new patient sessions, long and short therapy sessions, consultations, groups, medication management visits, and risk of harm evaluations.

### Variables

#### Demographic characteristics

Age was a continuous variable. Gender was coded as male (reference) and female. Preferred language was coded as English (reference), Spanish, and other (e.g., Haitian Creole and Port Creole/Cape). Insurance was coded as private (reference) and public. There were no missing data for the demographic data except for race/ethnicity. Although the data programmer did not report the percentage of missing data for race/ethnicity, variables with 10–40% missing data are often unusable without substantial imputation measures.^21^ Therefore, we did not extract the race/ethnicity variable, and as a result, it could not be included in the analysis.

#### Social vulnerability

We used the individual youth’s post office area (ZIP) code and matched it to the 2016 CDC Social Vulnerability Index (SVI) score.^22^ The CDC SVI ranks census areas (county subdivisions) based on 15 social factors, including race/ethnicity, language, poverty, and crowded housing on a scale as low (0 > 0.25), low to moderate (0.25 ≥ 0.50), moderate to high (0.50 ≥ 0.75), and high vulnerability (0.75 to 1).^22^ Data were missing for one patient.

#### Referral pathway

The standard policy at the Child & Adolescent Outpatient Psychiatry Center was to send clients to a screening clinic for a 15-minute MHI to determine needs before assigning clients to a provider for a full assessment and long-term care. This referral pathway was coded as MHI only. However, some patients were scheduled directly with a specific provider for long-term care. An internal clinician or social worker could schedule patients directly to individual providers. This referral pathway was coded as MHI + long-term care (reference). Details about who specifically scheduled patients directly to providers were not documented in medical records.

#### Patient engagement

Patient engagement was categorised as: initial (attended the first scheduled MHI visit: yes/no), delayed (number of rescheduled visits necessary for youth to attend the MHI), and sustained engagement (number of attended scheduled appointments, up to 24 visits, after youth completed the MHI). We set a cut-off of 24 visits, as only 23 of 815 (2.8%) youth had more than 24 appointments. Visits include therapy sessions, consultations, groups, medication management visits, and risk of harm evaluations. This cut-off aimed to account for the variability in specialty care, which extends beyond the typical 12–16 visits for evidence-based psychotherapy, and includes a range of treatment and support services.

### Analysis

We used descriptive statistics to report demographic data and chi-square and t-tests to examine the association between MHI only and MHI + long-term care pathway with initial, sustained, and delayed engagement. We used multiple logistic regression to assess the association between youth’s socio-demographic characteristics and referral pathway, controlling for age and gender. We conducted moderation analyses using multiple logistic and Poisson regressions to further examine the degree to which socio-demographic characteristics moderated the effect of the referral pathways on initial, sustained, and delayed engagement. Moderation analyses included interaction terms between the referral pathway and each moderator (preferred language, insurance type, and SVI), controlling for age and gender. All logistic regression results are presented as odds ratios.

### Ethics review

This study received ethical approval (BMC H-36037; UCLA IRB#20-002281) from the University of California, Los Angeles and the safety-net hospital in New England, USA.

## Results

The analysis included 815 new clients in 2016 with an average age of 12 years (standard deviation SD 3.98; range 3 to 18 years) (Table 1). Among these, just under half (377; 46.3%) were female, and most gave English as their preferred language (573; 70.3%), followed by Spanish (135; 16.6%). Most clients had public health insurance (685; 84.1%) and we observed high levels of social vulnerability (mean M SVI score 0.74; SD 0.27; range 0.007 to 0.97), with 66.5% (541) living in high vulnerability areas.

**Table 1.** Socio-demographic characteristics and engagement outcomes by referral pathway.

Variable	Total (*n* = 815)		MHI only pathway (*n* = 352)		MHI + long-term care pathway (*n* = 463)		Statistical test
	*n*	%	*n*	%	*n*	%	
Sex (female)	377	46.26	157	41.65	195	44.52	χ^2^ (1, 815) = 0.68
Preferred language							χ^2^ (2, 815) = 12.88*
English	573	70.31	267	46.60	306	53.40	
Spanish	135	16.56	40	29.63	95	70.37	
Other/unknown	107	13.13	45	42.06	62	57.94	
Health insurance							χ^2^ (1, 815) = 3.84*
Private insurance	130	15.95	46	35.38	84	64.62	
Public insurance	685	84.05	306	44.67	379	55.33	
Social vulnerability index							χ^2^ (3, 814) = 1.26
High social vulnerability	541	66.46	240	44.36	301	55.64	
Moderate to high social vulnerability	123	15.11	48	39.02	75	60.98	
Low to moderate social vulnerability	78	9.58	33	42.31	45	57.69	
Low social vulnerability	72	8.85	30	41.67	42	58.33	
Initial engagement	339	41.60	133	37.78	206	44.49	χ^2^ (1, 815) = 3.70∼
Ever engaged	493	60.49	182	51.70	311	67.17	χ^2^ (1, 815) = 20.01**

Variable	Total (*n* = 815)		MHI only pathway (*n* = 352)		MHI + long-term care pathway (*n* = 463)		Statistical test
	*M*	*SD*	*M*	*SD*	*M*	*SD*	
Delayed engagement (*n* = 493)	1.42	0.74	1.34	0.62	1.48	0.79	*t* (491) = 2.06*
Sustained engagement *n* = 493)	3.95	3.67	3.71	3.03	4.09	4.00	*t* (491) = 1.10
Age	12.33	3.98	11.86	3.95	12.69	3.97	*t* (813) = 2.98**
Social vulnerability	0.74	0.26	0.75	0.26	0.73	0.27	*t* (812) = −1.09

Note. *Significance level of *p* = < .05. **Significance level of *p* = < .001. ∼Trend towards significance at *p* = < .06. *M* refers to mean. *SD* refers to standard deviation.

### Referral pathway’s association with initial, delayed, and sustained engagement

#### Initial engagement

Of the 815 clients, less than half attended the first scheduled MHI visit (339; 41.6%). There was a trend towards fewer youth attending the first scheduled MHI appointment when referred to the screening clinic (37.8%) than when referred directly to a specific provider (44.5%; *p* = .054). Including rescheduled MHI appointments, 493 (60.5%) youth ever engaged in services over the observation period. Youth were less likely to ever engage if they were scheduled to the screening clinic (51.7%) than if they were scheduled to directly see a provider (67.2%; *p* < .001).

#### Delayed engagement

Of the 493 youth who ever engaged in care, most attended the first scheduled visit (339; 68.8%), followed by the second visit after missing the first (114; 23.1%). Only 8.1% (40) attended a visit after missing two scheduled appointments. Youth assigned to the screening clinic required fewer rescheduled appointments (M 1.34; SD 0.62) to engage in care than those referred directly to a provider (M 1.48; SD 0.79; *p* = .04).

#### Sustained engagement

Of the 493 clients who ever engaged in care, 24.8% (122) did not attend follow-up appointments, and about one in five attended only one (111; 22.5%), or five or more appointments (108; 21.9%). After the MHI, the mean number of follow-up appointments attended was 2.97 (SD 3.76; range 0 to 22), with no difference between screening clinic (M 3.12; SD 4.08) and direct referral to provider (M 2.73; SD 3.08).

### Association between youth’s socio-demographic characteristics and referral pathway

#### Preferred language

Youth whose preferred language was Spanish were less likely to be scheduled to the screening clinic (29.6%) than those whose preferred language was English (46.6%; Odds ratio OR 0.48; 95% confidence interval CI: 0.32, 0.73) (Table 2).

**Table 2.** Association between youth’s socio-demographic characteristics and referral pathway.

	Odds ratio	SE	Z ratio	*p*	95% confidence interval	
					Lower	Upper
Age	0.96	0.02	−2.37	.02*	0.92	0.99
Male	*Ref*					
Female	0.94	0.14	−0.44	.66	0.70	1.25
Preferred language						
English	*Ref*					
Spanish	0.48	0.10	−3.49	<.001*	0.32	0.73
Other/unknown	0.79	0.17	−1.06	.29	0.52	1.21
Type of insurance						
Private insurance	*Ref*					
Public insurance	0.65	0.13	−2.14	.03*	0.43	0.96
Social vulnerability index score	1.21	0.33	0.71	.48	0.71	2.07
Constant	1.42	0.45	1.11	.27	0.76	2.65

Note. The dependent variable in this logistic regression analysis is referral pathway coded as 0 = MHI + long-term care (direct referral to provider) and 1 = MHI only (screening clinic). The logistic regression controlled for age and gender. *Significance level of *p* = < .05. SE refers to the standard error. Z ratio is also known as a Z score or Z statistic.

#### Health insurance

Publicly insured youth were more likely to be scheduled in the screening clinic (44.7%) than those privately insured (35.4%; OR 0.56; 95% CI: 0.43, 0.96).

#### Social vulnerability

There were no differences in mean SVI scores between youth scheduled to the screening clinic versus those referred directly to a provider.

### Association between youth’s socio-demographic characteristics, referral pathway, and engagement

#### Initial engagement

Socio-demographic characteristics did not moderate the relationship between screening clinic and initial engagement. Youth who had private insurance (OR 2.21; 95% CI: 1.34, 3.66) and who lived in a low social vulnerability areas (OR 2.18; 95% CI: 1.10, 4.28) were more likely to attend the first scheduled MHI appointment. After accounting for these demographics, referral to the screening clinic was not significantly associated with first appointment attendance (OR 0.77; 95% CI: 0.51, 1.17) (Table 3).

**Table 3.** Association between youth’s socio-demographic characteristics, referral pathway, and initial engagement.

	Odds ratio	SE	Z ratio	*p*	95% confidence interval	
					Lower	Upper
Attendance at first scheduled MHI appointment						
Age	0.96	0.02	−2.57	.01*	0.92	0.99
Male	*Ref*					
Female	1.21	0.18	1.31	.19	0.91	1.63
Preferred language						
English	*Ref*					
Spanish	1.19	0.30	0.69	.49	0.73	1.94
Other/unknown	1.20	0.35	0.64	.52	0.68	2.11
Insurance type						
Private insurance	*Ref*					
Public insurance	2.21	0.57	3.1	.01*	1.34	3.66
Social vulnerability index						
High social vulnerability	*Ref*					
Moderate-high social vulnerability	1.20	0.32	0.67	.50	0.71	2.03
Low-moderate social vulnerability	1.04	0.34	0.12	.90	0.55	1.98
Low social vulnerability	2.18	0.75	2.25	.03*	1.10	4.28
MHI referral pathway						
MHI + long-term care	*Ref*					
MHI only	0.77	0.16	−1.21	.23	0.51	1.17
Interaction preferred language × referral pathway						
Spanish	1.43	0.61	0.83	.41	0.61	3.32
Other/unknown	0.48	0.23	−1.56	.12	0.19	1.21
Interaction insurance × referral pathway	0.67	0.29	−0.95	.34	0.29	1.53
Interaction social vulnerability × referral pathway						
Moderate-high social vulnerability	1.27	0.54	0.56	.57	0.55	2.94
Low-moderate social vulnerability	2.43	1.25	1.73	.08	0.89	6.66
Low social vulnerability	0.86	0.46	−0.29	.77	0.30	2.43
Constant	0.99	0.27	−0.04	.97	0.58	1.70
Ever attended a MHI appointment						
Age	0.97	0.02	−1.64	.10	0.93	1.01
Male	*Ref*					
Female	1.16	0.18	1.01	.32	0.87	1.57
Preferred language						
English	*Ref*					
Spanish	1.78	0.49	2.12	.03*	1.04	3.05
Other/unknown	1.20	0.35	0.64	.52	0.68	2.11
Insurance type						
Private insurance	*Ref*					
Public insurance	1.96	0.57	2.31	.02*	1.11	3.47
Social vulnerability index						
High social vulnerability	*Ref*					
Moderate-high social vulnerability	1.13	0.33	0.41	.68	0.64	1.99
Low-moderate social vulnerability	0.91	0.30	−0.29	.77	0.47	1.75
Low social vulnerability	2.69	1.17	2.27	.02*	1.14	6.31
MHI referral pathway						
MHI + long-term care	*Ref*					
MHI only	0.54	0.11	−1.96	.00*	0.36	0.81
Interaction preferred language × referral pathway						
Spanish	1.08	0.49	0.17	.87	0.44	2.65
Other/unknown	0.48	0.22	−1.58	.11	0.20	1.19
Interaction insurance × referral pathway	0.89	0.40	−0.28	.77	0.36	2.15
Interaction social vulnerability × referral pathway						
Moderate-high vulnerability	1.64	0.73	1.12	.26	0.69	3.93
Low-moderate vulnerability	3.43	1.87	2.26	.02*	1.18	9.98
Low vulnerability	0.50	0.30	−1.16	.25	0.16	1.61
Constant	2.06	0.59	2.53	.01	1.18	3.61

Note. The dependent variable in the logistic regression analysis for attendance at first scheduled MHI appointment is coded as 0 = did not attend first scheduled MHI appointment and 1 = yes, attended first scheduled MHI appointment. The dependent variable in the logistic regression analysis for ever engaged in care is coded as 0 = did not attend any scheduled MHI appointments and 1 = yes, attended at least 1 scheduled MHI appointment. The logistic regressions controlled for age and gender. *Significance level of *p* = < .05. SE refers to the standard error. Z ratio is also known as a Z score or Z statistic.

Youth who had private insurance (OR 1.96, 95% CI: 1.11, 3.47) and those who spoke Spanish (OR 1.78, 95% CI: 1.04, 3.05) were more likely to ever engage in care, however these factors did not moderate the relationship between screening clinic and ever attending a psychiatry visit (Table 3). SVI scores moderated the relationship between screening clinic and ever attending a psychiatry visit (OR 3.43; 95% CI: 1.18, 9.98). Youth living in the most vulnerable and least vulnerable areas were less likely to ever attend psychiatry services when referred to the screening clinic (Online Supplement Figure S1).

#### Delayed engagement

Socio-demographic characteristics did not moderate the relationship between referral pathway and number of rescheduled visits among youth who eventually engaged in care (Online Supplement Table S1).

#### Sustained engagement

Preferred language moderated the relationship between referral to the screening clinic and sustained engagement (β 0.55; 95% CI: 0.25, 0.83). Those whose preferred language was Spanish attended a greater number of follow-up appointments when they were referred to the screening clinic than when they were directly referred to a provider (Table 4 and Online Supplement Figure S2). Youth living in low vulnerability areas attended more appointments than youth living in high vulnerability neighbourhoods (β 0.28, 95% CI: 0.09, 0.48), however this factor did not moderate the relationship between being referred to the screening clinic and sustained engagement. Insurance type did not moderate the relationship between referral pathway and sustained engagement.

**Table 4.** Association between youth’s socio-demographic characteristics, referral pathway, and follow-up appointments after the MHI.

	β	SE	Z ratio	*p*	95% confidence interval	
					Lower	Upper
Age	−0.01	0.01	−0.98	.33	−0.02	0.01
Male	*Ref*					
Female	−0.11	0.05	1.98	.05*	000	0.21
Preferred language						
English	*Ref*					
Spanish	−0.22	0.10	−2.39	.02*	−0.40	−0.04
Other/unknown	−0.377	0.09	4.30	.00	0.21	0.55
Private insurance	*Ref*					
Public insurance	0.09	0.09	1.14	.25	−0.06	0.24
High social vulnerability	*Ref*					
Moderate-high social vulnerability	0.12	0.01	1.4	.16	−0.05	0.30
Low-moderate social vulnerability	−0.04	0.12	−0.36	.72	−0.28	0.19
Low social vulnerability	0.28	0.10	2.94	.00*	0.09	0.48
MHI + long-term care	*Ref*					
MHI only	−0.10	0.08	−1.18	.24	−0.26	0.07
Interaction preferred language × referral pathway						
Spanish	0.55	0.15	3.68	.00*	0.25	0.83
Other/unknown	−0.22	0.18	−1.23	.22	−0.57	0.13
Interaction insurance × referral pathway	−0.01	0.15	−0.61	.54	−0.38	0.20
Interaction social vulnerability × referral pathway						
Moderate-high social vulnerability	−0.16	0.15	−1.05	.29	−0.46	0.14
Low-moderate social vulnerability	−0.18	0.19	−0.94	.35	−0.56	0.20
Low social vulnerability	−0.15	0.18	−0.80	.43	−0.51	0.21
Constant	1.08	0.10	10.53	.00	0.88	1.28

Note. The dependent variable in this Poisson regression analysis is sustained engagement which is coded as the number of follow-up appointments after the MHI. The Poisson regression controlled for sex and gender. *Significance level of *p* = < .05. β refers to the beta coefficient. SE refers to the standard error. Z ratio is also known as a Z score or Z statistic.

## Discussion

This study examined the effects of two referral pathways on youth engagement in child psychiatry services in the largest safety-net hospital in New England, USA. We found that, in 2016, 58% of youth who were referred to the screening clinic or directly to a provider missed their first scheduled MHI appointment. Even after rescheduling, approximately 40% did not attend their MHI appointment. This level of non-attendance for the first appointment is almost twice as high than previously reported (33%).^2^ Our results demonstrate that low initial attendance in child psychiatric services continues to be a widespread issue, which has been associated with increased emergency service use, poor health outcomes, suicide, and premature mortality.^17,23,24^

We found that socio-demographic characteristics were associated with low engagement and MHI procedures. Thus, young people living in the most vulnerable areas in New England were less likely to ever engage in psychiatric care for the MHI. This is consistent with previous research which indicated that clients from high vulnerability neighbourhoods experience more challenges engaging in health care, often due to financial and logistical barriers.^25^ These barriers could have prevented youth from ever attending their MHI appointment. Similarly, young people with public insurance were less likely to attend their first MHI appointment compared to those with private insurance. This is also consistent with national data suggesting that people with public insurance have lower engagement with mental health care despite greater need,^26^ often due to service costs.^26,27^ We further found that people with public insurance were more likely to be referred to the MHI only pathway than privately insured youth, possibly due to third-party reimbursement policies.^10,18^ Clients with public insurance are less likely to successfully schedule primary care appointments^26^ which is often required for specialty care. Administrative staff at the Child & Adolescent Outpatient Psychiatry Center may have used information about external referrals, such as from primary care,^19^ and youth’s insurance type to determine which clients could be referred to the MHI only or the MHI + long-term care pathways, as private insurance did not reimburse the MHI only pathway.

These findings point to an urgent need for psychiatric services to evaluate organisational processes to mitigate clinic-based barriers and proactively engage youth who are at higher risk of not attending their initial MHI appointment. For example, we found that Spanish-speaking youth were less likely to be referred to the MHI only pathway and were more likely to engage in psychiatric care, which contradicted our initial hypothesis. Our findings might reflect the safety-net hospital’s availability of bilingual Spanish-speaking providers,^20^ who could conduct the MHI in Spanish and may have helped Spanish-speaking clients overcome linguistic barriers and improve engagement in care.^28,29^

MHI procedures often confuse clients,^17^ with differences in administrative workflow, service availability, and third-party reimbursement for specific visit types.^1^ This variation can reduce engagement in psychiatry services. We found that youth were less likely to ever attend a MHI appointment when referred through the MHI only pathway. We also found that youth in the MHI + long-term care referral pathway had more rescheduled MHI appointments than youth in the MHI only pathway. These findings suggest that referral pathway type may influence appointment attendance and rescheduling, potentially due to differences in administrative workflow decisions unique to each pathway;^1^ however, further research is needed to examine these pathways in greater detail.

Finally, we found that clients attended a total of four sessions on average, which is consistent with attendance rates in other mental health care settings.^4,16^ Spanish speakers attended slightly more follow-up appointments on average when referred to the MHI only pathway. This implies that while having a Spanish-speaking provider in the MHI + long-term care pathway may be beneficial, it may not address all barriers to sustained engagement.^28^ Screening clinics that conduct the MHI should evaluate key factors such as client’s diagnosis, cultural background, structural barriers, and experiences of discrimination, racism, and trauma.^11–13,18^ These factors are critical for effective provider-client matching, ensuring that client needs align with provider skills and training, and can significantly impact sustained engagement in follow-up care. In addition to addressing clients’ language needs,^11,29^ further research is needed to better understand how differences in referral pathway processes, such as length of visit, quickly addressing structural or social needs when referred to other public sectors, and making referrals, can improve engagement in child psychiatric services.

### Study limitations

Our dataset did not allow assessing participants’ racial/ethnic identity and we were thus not able to investigate racial and ethnic disparities in engagement with psychiatric services. Due to the limited nature of our dataset, the reasons why youth terminated treatment or which providers scheduled clients directly to individual providers cannot be assessed. This limits our understanding of potential workforce availability and workflow decisions that may impact engagement. We did not find that SVI scores predicted the likelihood of being referred to the screening clinic or to a direct provider, possibly due to the low variability in scores, as over 80% of participants lived in moderate to high vulnerability areas. Finally, our study was conducted in one hospital only and findings are therefore not generalisable.

## Conclusions

Our findings suggest that the MHI + long-term care pathway may be more effective at engaging youth in psychiatry services than the MHI only pathway. However, there is a need for further research to better understand the content and focus of each of type of MHI referral pathway, and organisational decision making. Psychiatry services should identify and address structural barriers, and consider adopting new procedures, such as the use of interdisciplinary teams that could engage youth during MHI appointments and reduce treatment dropout. If psychiatry services can keep clients engaged in care, it might help the nation offset a loss of revenue of $150 billion annually for missed appointments^30^ and improve mental health outcomes for youth in the USA.

## Supplemental Material


Supplemental Material - Engagement in child psychiatry department appointments: An analysis of electronic medical records in one safety-net hospital in New England, USA
Supplemental Material for Engagement in child psychiatry department appointments: An analysis of electronic medical records in one safety-net hospital in New England, USA by Yesenia Aguilar Silvan, Lisa R Fortuna, Andrea E Spencer and Lauren C Ng in Journal of Health Services Research & Policy.
