# Supplementary material for: Engagement in child psychiatry department appointments: An analysis of electronic medical records in one safety-net hospital in New England, USA
Source: J Health Serv Res Policy. 2025 Jan 16;30(2):79–88. doi: 10.1177/13558196241311712 (PMC11877985; doi:10.1177/13558196241311712)
Supplement: Supplemental Material - Engagement in child psychiatry department appointments: An analysis of electronic medical records in one safety-net hospital in New England, USA [file sj-pdf-1-hsr-10.1177_13558196241311712.pdf]

Journal of Health Services Research & Policy
Engagement in child psychiatry department appointments: An analysis of electronic medical records in one safety-net hospital in New England, USA
Aguilar Silvan Y, Fortuna LR, Spencer AE and Ng LC

Online Supplement

Figure S1. Association between social vulnerability index score, referral pathway, and ever attending a MHI appointment

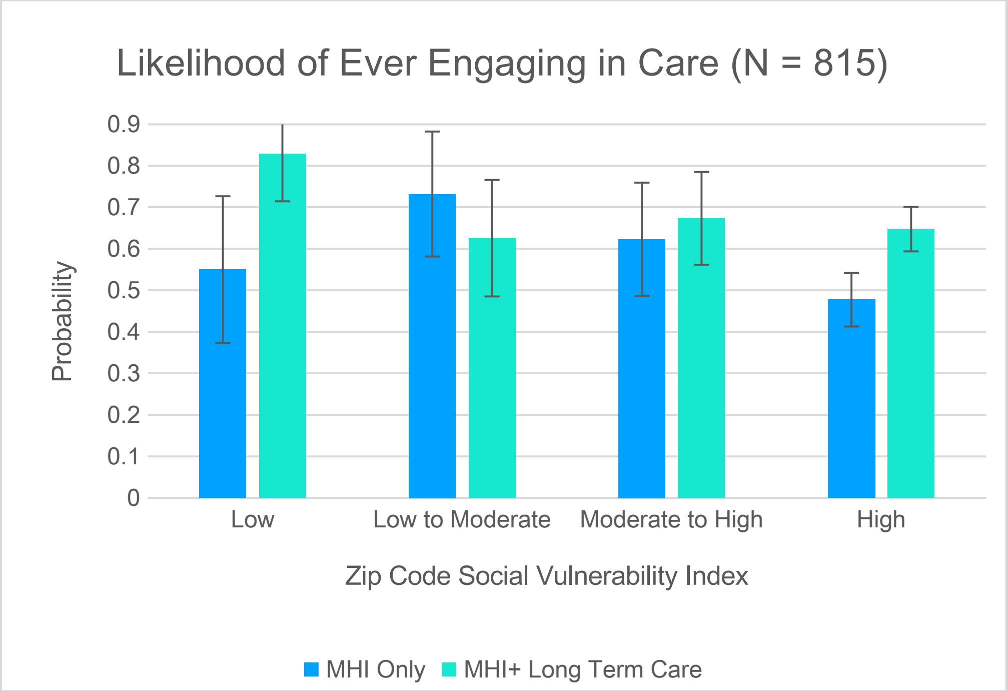

Note. Error bars = 95% Confidence Interval

Journal of Health Services Research & Policy
Engagement in child psychiatry department appointments: An analysis of electronic medical records in one safety-net hospital in New England, USA
Aguilar Silvan Y, Fortuna LR, Spencer AE and Ng LC

Figure S2. Association between preferred language, referral pathway, and follow-up appointments

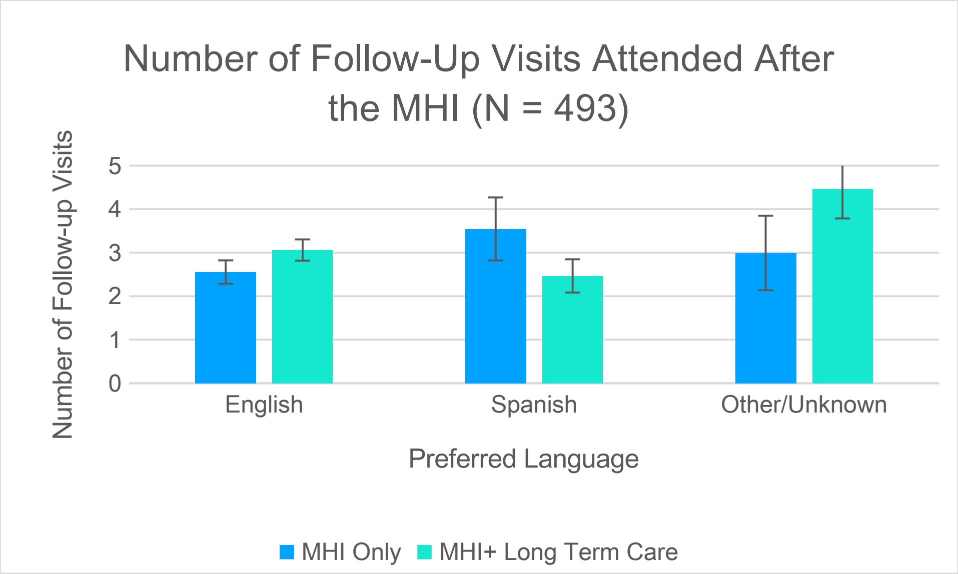

Note. Error bars represent 95% Confidence Interval

Table S1. Association between youth's socio-demographic characteristics, referral pathway, and rescheduled sessions

					95% Confidence interval	
	β	SE	Z Ratio	p	Lower	Upper
Age	0.01	0.01	0.56	0.57	-0.01	0.03
Male	Ref.					
Female	-0.03	0.08	-0.45	0.65	-0.19	1.17
English	Ref.					
Spanish	0.09	0.12	0.78	0.43	-0.14	0.32
Other/unknown	-0.05	0.15	-0.32	0.75	-0.34	0.24
Private insurance	Ref.					
Public insurance	-0.14	0.13	-1.15	0.25	-0.39	0.10
High social vulnerability	Ref.					
Moderate-high social vulnerability	-0.07	0.13	-0.54	0.59	-0.33	0.19
Low-moderate social vulnerability	-0.00	0.17	-0.03	0.98	-0.33	0.32
Low social vulnerability	-0.01	0.15	-0.06	0.95	-0.31	0.29
MHI + long-term care	Ref.					
MHI only	-0.12	0.12	-1.01	0.31	-0.35	0.11
Language x Referral pathway						
Spanish	-0.11	0.22	-0.49	0.63	-0.53	0.32
Other/unknown	0.00	0.27	0.01	0.99	-0.53	0.54
Insurance x Referral pathway	0.12	0.22	0.57	0.57	-0.30	0.55
Social vulnerability x Referral Pathway						
Moderate-high	0.11	0.22	0.48	0.63	-0.33	0.55

Journal of Health Services Research & Policy
Engagement in child psychiatry department appointments: An analysis of electronic medical records in one safety-net hospital in New England, USA
Aguilar Silvan Y, Fortuna LR, Spencer AE and Ng LC

					95% Confidence interval	
	β	SE	Z Ratio	p	Lower	Upper
social vulnerability						
Low-moderate social vulnerability	0.02	0.26	0.06	0.95	-0.49	0.53
Low social vulnerability	-0.00	0.28	-0.02	0.99	-0.56	0.56
Constant	0.36	0.15	2.42	0.02	0.07	0.65

Note: The dependent variable in this Poisson regression analysis is delayed engagement which is coded as the number of rescheduled appointments necessary to conduct the MHI. The Poisson regression controlled for age and gender. * Significance level of p = < 0.05. β refers to the beta coefficient. SE refers to the standard error. Z ratio is also known as a Z score or Z statistic.
